# Pathogen enrichment sequencing (PenSeq) enables population genomic studies in oomycetes

**DOI:** 10.1111/nph.15441

**Published:** 2018-10-05

**Authors:** Gaetan J. A. Thilliez, Miles R. Armstrong, Tze‐Yin Lim, Katie Baker, Agathe Jouet, Ben Ward, Cock van Oosterhout, Jonathan D. G. Jones, Edgar Huitema, Paul R. J. Birch, Ingo Hein

**Affiliations:** ^1^ Cell and Molecular Sciences The James Hutton Institute Errol Road, Invergowrie Dundee DD2 5DA UK; ^2^ Division of Plant Sciences at the James Hutton Institute School of Life Sciences University of Dundee Dundee DD2 5DA UK; ^3^ Information and Computational Sciences The James Hutton Institute Dundee DD2 5DA UK; ^4^ The Sainsbury Laboratory Norwich Research Park Norwich NR4 7GJ UK; ^5^ The Earlham Institute Norwich Research Park Norwich NR4 7UH UK; ^6^ University of East Anglia Norwich Research Park Norwich NR4 7TJ UK

**Keywords:** avirulence, PenSeq, *Phytophthora capsici*, *Phytophthora infestans*, population genomics, RXLR effectors, virulence

## Abstract

- The oomycete pathogens *Phytophthora infestans* and *P. capsici* cause significant crop losses world‐wide, threatening food security. In each case, pathogenicity factors, called RXLR effectors, contribute to virulence. Some RXLRs are perceived by resistance proteins to trigger host immunity, but our understanding of the demographic processes and adaptive evolution of pathogen virulence remains poor.
- Here, we describe PenSeq, a highly efficient enrichment sequencing approach for genes encoding pathogenicity determinants which, as shown for the infamous potato blight pathogen *Phytophthora infestans*, make up < 1% of the entire genome.
- PenSeq facilitates the characterization of allelic diversity in pathogen effectors, enabling evolutionary and population genomic analyses of *Phytophthora* species. Furthermore, PenSeq enables the massively parallel identification of presence/absence variations and sequence polymorphisms in key pathogen genes, which is a prerequisite for the efficient deployment of host resistance genes.
- PenSeq represents a cost‐effective alternative to whole‐genome sequencing and addresses crucial limitations of current plant pathogen population studies, which are often based on selectively neutral markers and consequently have limited utility in the analysis of adaptive evolution. The approach can be adapted to diverse microbes and pathogens.

## Abstract 2

See also the Commentary on this article by Kale, **221**: 1177–1179.

## Introduction

The oomycete pathogens *Phytophthora infestans* and *P. capsici* are widespread and economically significant threats to global crop production. *Phytophthora infestans* causes late blight disease in Solanaceae plants, including potato and tomato, and led to the Irish potato famine in the mid‐1840s (Birch *et al*., 2012). *Phytophthora capsici* shares with *P. infestans* the common hosts tomato and *Nicotiana benthamiana*, but infects, in addition to some Solanaceae, a number of plants within the Cucurbitaceae and Fabaceae families (Lamour *et al*., 2012a). Both *Phytophthora* species are very destructive and can adapt rapidly to new selection pressures imposed by modern agriculture (Rocha‐Castro *et al*., 2014; Fry *et al*., 2015). As shown for *P. infestans*, the migration of new genotypes can result in major population shifts. This is exemplified by the rapid emergence of the *P. infestans* lineage 13_A2 in Europe, which came to dominate the UK pathogen population within 3 yr of its arrival (Cooke *et al*., 2012). Such dramatic change is often marked by the spread of clonal, asexual lineages (Fry *et al*., 2015), usually tracked through traditional population genetic studies of a few selectively neutral markers.

On a molecular level, pathogen avirulence or virulence to naturally occurring or deployed host disease resistances is determined by effectors, which, for both *P. infestans* and *P. capsici*, may contain a signal peptide (SP) followed by the canonical arginine (Arg)‐any amino acid‐leucine (Leu)‐Arg (RXLR) domain (Birch *et al*., 2006; Hein *et al*., 2009; Lamour *et al*., 2012a). The effector recognition‐based inducible defence response is often governed by nucleotide‐binding, leucine‐rich repeat (NLR) disease resistance proteins, and the resulting incompatibility is known as effector‐triggered immunity (ETI) (Jones & Dangl, 2006; Dodds & Rathjen, 2010; Jones *et al*., 2016). Despite an increasing molecular understanding of the mechanisms that govern pathogen virulence or avirulence, our understanding of the demographic processes and adaptive evolution of virulence of *P. infestans* remains poor.

The repeat‐rich, 240‐megabase (MB) genome of the *P. infestans* strain T30‐4 and the 64‐MB genome of the partially inbred *P. capsici* line LT1534 have provided an overview of the genomic organization of effectors (Haas *et al*., 2009; Lamour *et al*., 2012b). In contrast with core orthologous genes, RXLRs are predominantly found in gene‐sparse, repeat‐rich regions, which has been proposed to facilitate their fast evolution (Haas *et al*., 2009; Raffaele *et al*., 2010; Lamour *et al*., 2012b). Various computational algorithms have been developed to predict RXLR effector genes in oomycete genomes (Bhattacharjee *et al*., 2006; Whisson *et al*., 2007; Win *et al*., 2007; Haas *et al*., 2009). The number of predicted RXLRs in different oomycetes varies considerably, with 563 in the *P. infestans* genome (Haas *et al*., 2009), 516 in *P. capsici* (J. Jupe *et al*., 2013), 531 in *P. ramorum*, 672 in *P. sojae* (Tyler *et al*., 2006) and 149 in *Hyaloperonospora arabidopsidis* (Win *et al*., 2007; Baxter *et al*., 2010). Compared with the overall genome sizes of these oomycetes, RXLR effector repertoires typically are encoded by < 1% of a given pathogen genome.

The availability of genome‐wide sequence information from plant pathogens has given rise to population genomics, a form of population genetics based on large‐scale genotyping (Grünwald *et al*., 2016). This study describes the development and utilization of targeted Pathogen enrichment Sequencing (PenSeq) for secreted proteins, including RXLR effectors which are required for disease. The development of PenSeq is a critical and logical next step to understand the evolutionary forces that govern plant–pathogen coevolution in a targeted and therefore cost‐effective manner. An understanding of pathogen diversity on a molecular level is a prerequisite for the development and achievement of more durable resistance in crops with wide‐reaching consequences for food security. The method has broad applications and can be adapted to diverse microbes and pathogens across a wide host spectrum. The secretome and, particularly, the RXLRs are of interest, as adaptive evolutionary changes are most likely to occur in these genes in response to host recognition responses (Albert *et al*., 2007; Hodges *et al*., 2007; Cronn *et al*., 2012). Compared with whole‐genome sequencing, the sequencing of enriched samples offers the benefits of achieving sufficient read depth of targeted genes to facilitate the accurate identification of sequence polymorphisms (Parla *et al*., 2011; Saintenac *et al*., 2011). Such deep sequencing is also important to uncover copy number variation (CNV) in multigene families with high allelic variance (Lighten *et al*., 2014). As shown in plants, target enrichment sequencing has proven to be an effective tool to aid the annotation of genomes, to map new traits and to be utilized as a diagnostic tool to study the presence/absence as well as sequence variations (F. Jupe *et al*., 2013; Van Weymers *et al*., 2016; Chen *et al*., 2018; Jiang *et al*., 2018).

## Materials and Methods

### *Phytophthora* mycelial growth and DNA extraction

*Phytophthora capsici* was grown on pea broth medium at 28°C for 3 d; *P. infestans* was grown on pea broth medium at 20°C for 7 d. Mycelia were harvested, dried using a vacuum pump and stored at −80°C until DNA extraction. DNA extraction from mycelia was conducted using the cetyltrimethylammonium bromide (CTAB) method described by Wangsomboondee & Ristaino (2002).

### Bait design

Biotinylated, RNA‐derived baits of 120 nucleotides (nt) in length were designed for targeted genes using end‐to‐end tilling and were manufactured by MYcroarray (MYbaits; Ann Arbor, MI, USA). The library contains 18 348 baits in total (see Jouet *et al*., 2019), 7296 of which were designed to target *P. infestans* and *P. capsici* genes. Of the latter, 3729 target *P. infestans* genes with gene identifiers (PITG, including predicted RXLRs and other genes of interest), 24 were specifically designed to enable the enrichment of additional RXLRs identified in the UK isolate 3928A, and 10 target other *P. infestans* genomic loci with no known PITG identifier. Similarly, 2531 baits were designed to target *P. capsici* predicted RXLRs, and 1002 baits to target *P. capsici* predicted Crinklers (CRNs).

### Target enrichment sequencing

DNA from all *Phytophthora* isolates was sheared with an M220 Focused‐ultrasonicator (Covaris, Woburn, MA, USA). The following conditions were used on 50 μl containing 1 μg of DNA to obtain 500‐bp‐long fragments: peak, 50 W; 200 cycles per burst; duty factor of 20 for 60 s. The size of the fragment was checked on a 2100 Bioanalyzer (Agilent Technologies, Santa Clara, CA, USA). After library preparation, performed according to J. Jupe *et al*. (2013) and F. Jupe *et al.* (2013), a Qubit instrument (Thermofisher, Waltham, MA, USA) was used to quantify DNA from each isolate. Equimolar amounts of DNA from the 12 individually barcoded samples were pooled to obtain 500 ng of total DNA. Enrichment was performed employing the protocol described in the Mybait user book v.2.3 (MYcroarray) using conditions which allow for more interspecific hybridization. The enrichment hybridization was incubated for 37 h. The post‐capture amplification was performed with Herculase II polymerase (Agilent Technologies). Paired‐end sequencing of the enriched pool libraries was conducted on an Illumina MiSeq platform using 2 × 300‐bp chemistry. Cutadapt was used for adapter trimming and quality trimming (Martin, 2011), and FastQC (v.0.10.0) was used to generate a quality control report of MiSeq reads (Andrews, 2010), as described in J. Jupe *et al*. (2013) and F. Jupe *et al*. (2013).

### Computational analyses

The computational analyses for single nucleotide polymorphism (SNP) calling, nucleotide diversity and heterozygosity study, *de novo* RXLR predictions and relative gene expression analysis are detailed in Supporting Information Methods S1.

### PCR‐based validation of PenSeq‐predicted presence/absence variations

PCR primers were designed for 10 RXLR genes to confirm their presence/absence in the six isolates (Methods S1).

## Results

Biotinylated RNA baits for selected *P. infestans* and *P. capsici* genes were used for target enrichment of genomic DNA. We refer to this new method as PenSeq following the nomenclature used for Resistance gene enrichment Sequencing (RenSeq) (F. Jupe *et al*., 2013). The probe library was designed to capture genes that encode *Phytophthora* secreted proteins, including RXLR effectors (Table S1). These include annotated genes from the *P. infestans* T30‐4 reference genome, which are denoted with PITG identifiers (Haas *et al*., 2009), and novel RXLRs identified in isolate 3928A (Cooke *et al*., 2012), referred to as PiUK3928A genes. We also targeted *P. capsici* predicted RXLRs, annotated as PcRXLR (Lamour *et al*., 2012b; F. Jupe *et al*., 2013), and predicted CRNs (Lamour *et al*., 2012b; Stam *et al*., 2013).

Target enrichment was conducted simultaneously for genomic DNA from six individually barcoded *P. infestans* and six *P. capsici* isolates. Post‐enrichment samples were sequenced on a single lane of Illumina MiSeq. The *P. infestans* selection comprised diverse genotypes, including a copy of the reference strain T30‐4 (Haas *et al*., 2009), and the isolates 88069 (Whisson *et al*., 2007), EC1‐C7, 3928A (representing genotype 13_A2; Cooke *et al*., 2012), 110059 (genotype US23) and 110153 (genotype US24). The *P. capsici* isolates included the reference strain LT1534 (Lamour *et al*., 2012b) and isolates LT123, PC204, LT6535, Y006 and Q108. Following adapter trimming and quality control, 39 630 942 high‐quality post‐enrichment reads (‘PenSeq reads’) were retained for downstream analysis. Of these, 10 487 858 reads originated from *P. infestans* and 29 143 084 from *P. capsici* isolates (Table S2).

### PenSeq reads have a high ‘on‐target’ rate when mapped against the *Phytophthora* reference genomes

The percentage of mapped reads on‐target was calculated as the proportion of PenSeq‐derived reads mapping to the respective reference genome at positions that are associated with baits. These include bait design sequences for which probes and targets are 100% identical and putative bait target sites that exhibit a minimum of 80% sequence identity with corresponding baits (F. Jupe *et al*., 2013). Intersecting these regions (plus 1000 bp upstream and downstream) against the mapped PenSeq reads yields the number of on‐target reads.

For the *P. infestans* T30‐4 genome, 1457 534 high‐quality PenSeq reads were obtained. Of these, 1136 644 reads could be mapped to the reference genome at a 1% mismatch rate (Table 1). Of the latter, 427 792 reads (37.64% of all mapped reads) corresponded to bait design sites with 100% sequence identity, and 573 168 reads (50.43%) to bait binding sites with a minimum of 80% sequence identity. Increasing the mismatch rate to 5% enabled the mapping of 1349 756 of the 1457 534 PenSeq reads to the reference. Of these, 484 648 (35.91% of mapped reads) corresponded to bait design sites and 654 139 (48.46%) to bait binding sites.

**Table 1:** Proportion of on‐target reads for the T30‐4 and LT1534 reference genomes

MM	Read input	Reads mapped	Bait design sites (100% sequence identity)			Bait binding sites (min 80% sequence identity)		
			Reads	Percentage of total reads (%)	Percentage of mapped reads (%)	Reads	Percentage of total reads (%)	Percentage of mapped reads (%)
T30‐4								
1%	1457 534	1136 644	427 792	29.35	37.64	573 168	39.32	50.43
2%	1457 534	1262 455	461 847	31.69	36.58	621 805	42.66	49.25
5%	1457 534	1349 756	484 648	33.25	35.91	654 139	44.88	48.46
LT1534								
1%	2085 062	971 963	432 549	20.75	44.50	646 163	30.99	66.48
2%	2085 062	1231 333	520 357	24.96	42.26	831 119	39.86	67.50
5%	2085 062	1475 423	619 139	29.69	41.96	990 977	47.53	67.17

The on‐target rate for PenSeq reads (Read input) is shown for the *Phytophthora infestans* T30‐4 genome and the *P. capsici* LT1534 reference genome. High‐quality PenSeq reads are mapped to the respective reference isolates under different mismatch (MM) conditions. A 1% mismatch rate allows for a maximum of one sequence polymorphism in a 100‐bp target sequence, whereas 2% and 5% allow for up to two and five polymorphisms, respectively.

Interestingly, PenSeq reads derived from EC1‐C7, 3928A, 110059 and 110153, when mapped against the T30‐4 reference genome at a 1% mismatch rate, had similar if not slightly higher on‐target rates, ranging from 51.98% for isolate 3928A to 61.71% for isolate 110059 (Table S2). Comparable on‐target rates were achieved for the bait design and putative bait binding sites in *P. capsici* when mapping LT1534‐derived PenSeq reads to the *P. capsici* reference genome. The percentage of PenSeq reads that mapped to the bait design sites at a 1% mismatch rate accounted for 44.50% of all mapped reads, and increased to 66.48% for the on‐target rate for putative bait binding sites (Table 1).

Considering the genome sizes of *P. infestans* (240 MB) and *P. capsici* (64 MB), and the gene space that was specifically enriched in both oomycetes (0.436 MB in *P. infestans* and 0.394 MB in *P. capsici;* Table S1), the achieved on‐target rates at a 1% mismatch rate correspond to an average 308‐fold enrichment for *P. infestans* and 101‐fold enrichment for *P. capsici* isolates.

### Targeted *Phytophthora* genes are highly represented by PenSeq reads

**Figure 1:**
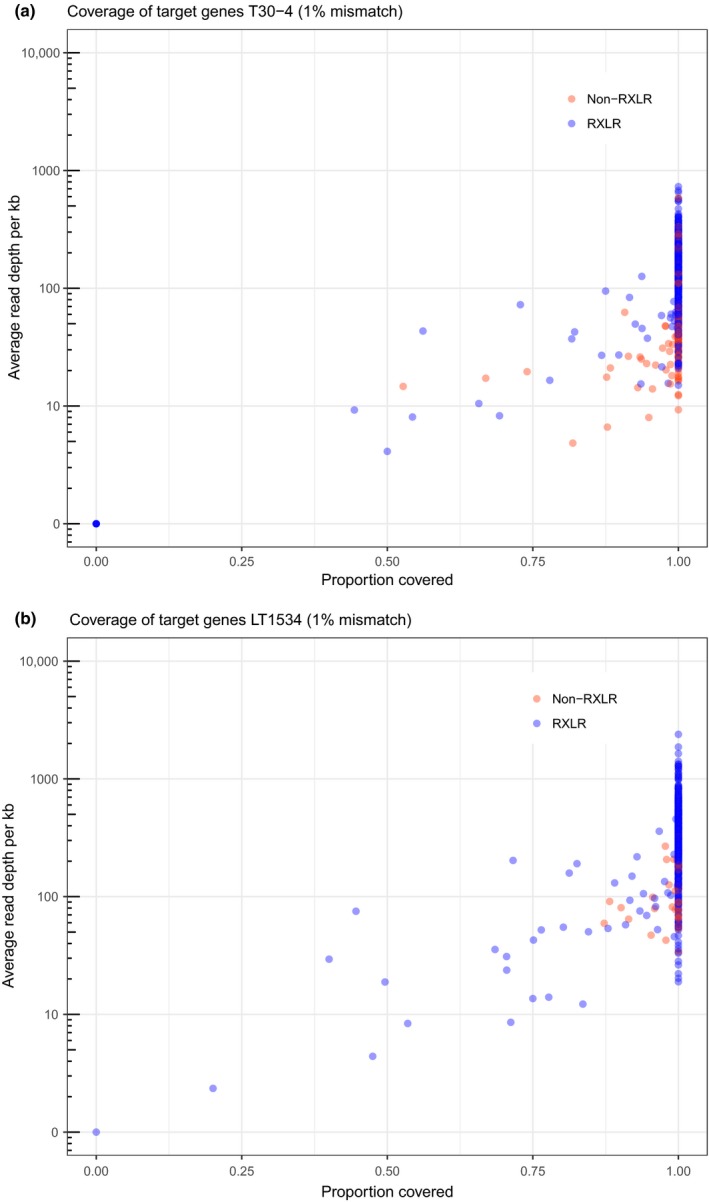
Representation of target gene coverage in (a) *Phytophthora infestans* reference strain T30‐4 and (b) *P. capsici* reference strain LT1534 at a high‐stringent, 1% mismatch mapping rate. The *x*‐axis represents the percentage gene coverage of RXLRs (blue) and non‐RXLR target genes (red), which ranges from 0 (not covered) to 1 (100% sequence representation with PenSeq reads). The *y*‐axis reveals the average read depth per kb of the target genes.

The coverage of RXLR and non‐RXLR target genes was first evaluated for the reference genomes T30‐4 and LT1534 as the baits were designed from these genome sequences (Table S1). PenSeq analysis for the T30‐4 reference genome yielded 1457 534 high‐quality reads (Tables 1, S2). These reads were mapped against the T30‐4 genome at high stringency, allowing for a 1% mismatch rate (Fig. 1a), and the read coverage of regions corresponding to the 579 targeted genes was determined. Medium‐stringent (2% mismatch rate) and low‐stringent (5% mismatch rate) mapping conditions were also assessed to ascertain the level of gene coverage achieved in the multiplexing approach utilized (Fig. S1).

As expected, the number of target genes that were fully covered by PenSeq reads correlated with the read mapping stringencies applied. For the T30‐4 genome, 505 genes were fully represented at 1%, 522 at 2% and 529 at 5% mapping mismatch rates. This represented, at the most stringent mapping condition (1% mismatch rate), > 87% of all targeted genes that are fully covered by PenSeq reads. The remaining, partially covered genes had, on average, a read representation of between 88.43% (at 1% mismatch rate) and 82.76% (at 5% mismatch rate) of the full‐length genes (Figs 1, S1). Unexpectedly, 16 RXLR effector sequences were not covered by T30‐4‐derived PenSeq reads, but were represented in at least two other isolates, such as 3928A and EC1‐C7, which yielded similar numbers of high‐quality PenSeq reads (Tables 2, S2). This is indicative of a presence/absence polymorphism in this specific T30‐4 isolate analysed, rather than an ascertainment problem or bait failure, as the DNA from all isolates was multiplexed and enriched simultaneously.

**Table 2:** Sequence coverage of effectors that are absent from the reference genomes across diverse isolates

Effector	Sequence coverage in isolates (0 = 0%–1 = 100%)					
*Phytophthora infestans*	T30‐4	3928A	88069	110059	110153	EC1‐C7
PITG_04085	0	1	1	1	1	1
PITG_04089	0	0.92	0.76	0.96	0.60	0.63
PITG_04097	0	1	1	1	1	1
PITG_04099	0	1	1	1	1	1
PITG_04182	0	0.97	1	1	0	1
PITG_04279	0	0	1	1	1	1
PITG_12010	0	0	1	1	1	1
PITG_16282	0	1	1	1	1	1
PITG_16283	0	1	1	1	1	1
PITG_16285	0	1	1	1	1	1
PITG_19800	0	1	1	1	1	1
PITG_21107	0	1	0	0	0	1
PITG_21778	0	1	1	1	1	1
PITG_22724	0	1	1	1	1	1
PITG_22727	0	1	1	1	1	1
PITG_23011	0	1	0	1	0	0

Effector	Sequence coverage in isolates (0 = 0%–1 = 100%)					
*P. capsici*	LT1534	LT123	Pc204	LT6535	Y006	Q108
PcRXLR005	0	1	0.32	0.30	0.36	1

The sequence coverage of 16 *Phytophthora infestans* effectors that were not enriched in the T30‐4 genome is shown for the *P. infestans* isolates 3928A (13_A2), 88069, 110059 (US23), 110153 (US24) and EC1‐C7 (EC1). The sequence coverage of the *P. capsici* effectors that were not identified in the LT1534 reference gene is shown for the *P. capsici* isolates LT123, Pc204, LT6535, Y006 and Q108. The sequence coverage with PenSeq‐derived reads is shown as a percentage, ranging from 0 (no coverage) to 1 (full coverage).

To independently validate the observation that effectors are absent in this T30‐4 isolate, which has been maintained since before the genome sequence was released in 2009 (Haas *et al*., 2009), as well as in other isolates where predicted, we conducted PCR amplifications of selected genes. Using the core gene *Avr3a* (PITG_14371) as a positive control, PCRs were conducted for nine additionally randomly selected genes from the 16 PITGs (Fig. S2). With the exception of PITG_22727, which failed to yield a reproducible amplicon for all isolates, the absence of effectors PITG_04097, PITG_04099, PITG_04182, PITG_12010, PITG_16282, PITG_16283, PITG_16285 and PITG_19800 in the isolate T30‐4 could be independently confirmed by PCR. Similarly, the absence of PITG_04182 in the US24 isolate 110153 and PITG_12010 in 3928A was also independently confirmed. It is worthwhile noting that the PCR products for effectors PITG_04097 and PITG_16283 were weaker when compared with other effectors, despite utilizing the same amount of template DNA for all amplifications. This highlights the potential ambiguity of PCR‐based presence/absence analysis and further highlights the efficacy and advantages of PenSeq.

In total, 2 085 062 PenSeq reads were obtained for the *P. capsici* isolate LT1534 (Tables 1, S2) and mapped against the published reference genome (Lamour *et al*., 2012b) at the same mismatch rates as used for the T30‐4 target enrichment analysis (Figs 1b, S1). At a 1% mismatch rate, 517 target genes (representing > 90% of all selected genes) were fully represented by PenSeq reads and 56 were partially covered, with an average of 85.02% sequence representation of the full‐length genes. PenSeq reads from LT1534 were only missing for one gene, PcRXLR005. However, PcRXLR005 was fully represented in *P. capsici* isolates LT123 and Q108 at a 1% mismatch rate (Table 2). Allowing for a 2% mapping mismatch rate yielded 559 genes that were fully represented and 15 that were partially covered, with an average sequence representation of 85.01% (Fig. S1). At a 5% mismatch rate, 572 target genes were fully represented by PenSeq reads and only two genes were partially covered, with an average sequence representation of 76.04% (Fig. S1).

This analysis was widened to include the nonreference *P. infestans* and *P. capsici* isolates. At a 1% mismatch rate, the average representation of all target genes by PenSeq‐derived reads was 90.97% across the *P. infestans* and *P. capsici* isolates (Fig. S3). This provides evidence that the gene‐specific probes performed effectively during the hybridization and that sufficient sequencing coverage was achieved across diverse *Phytophthora* species and isolates.

### PenSeq enables nucleotide diversity and heterozygosity evaluation of RXLR and non‐RXLR encoding genes in *P. infestans*

**Figure 2:**
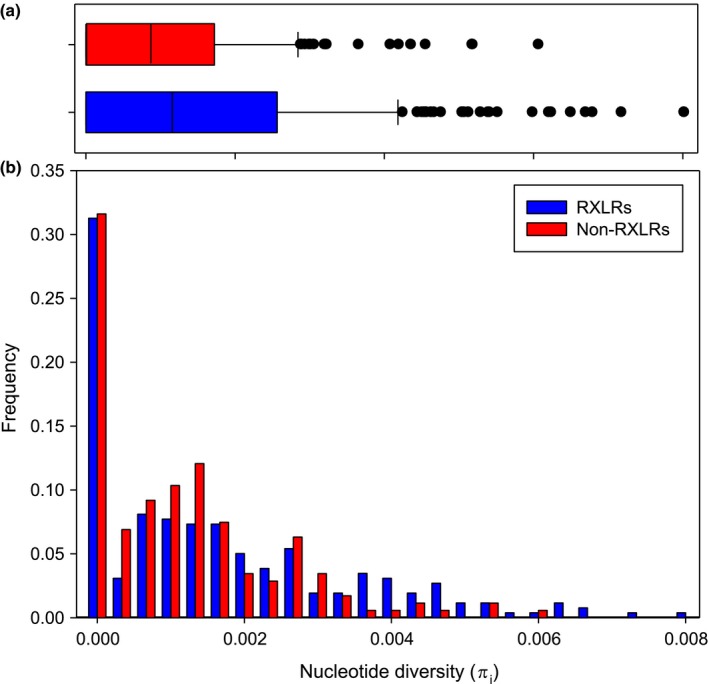
(a) Comparative nucleotide diversity study of 259 RXLRs and 174 non‐RXLRs across six *Phytophthora infestans* isolates. The mean (± SE) nucleotide diversity of the RXLRs (1.63 × 10^−3^ (± 1.10 × 10^−4^)) is significantly higher than that of non‐RXLRs (1.14 × 10^−3^ (± 0.90 × 10^−4^)) (Mann–Whitney test, *W* = 58962.5, *P* = 0.0282 (adjusted for ties)). (b) Nucleotide diversity calculated for 433 *P. infestans* PITGs, with 259 RXLRs (blue) and 174 non‐RXLRs (red) across six *P. infestans* isolates. An outlier analysis identified seven PITGs (PITG_06432, PITG_07947, PITG_10396, PITG_11484, PITG_13018, PITG_14986 and PITG_18325) which all encode for RXLRs and display significantly elevated nucleotide diversity (π_i_), indicative of non‐neutral evolution. The difference between the RXLR and non‐RXLR distributions of π_i_ is driven by these seven outlier loci, without which the difference in π_i_ is no longer significant (Mann–Whitney test, *W* = 55952.5, *P* = 0.0802 (adjusted for ties)).

*Phytophthora infestans* RXLR and non‐RXLR encoding genes that were included in the bait library design (Table S1) were assessed for their representation by PenSeq reads in all six isolates. Of the 579 targeted *P. infestans* genes, 433 genes were present in all six isolates and comprised 259 ‘core’ RXLRs and 174 non‐RXLRs. As a result of the high gene coverage achieved through enrichment sequencing (Fig. 1a), the nucleotide diversity and heterozygosity could be calculated for these genes. A comparison of the nucleotide diversity between RXLR and non‐RXLR coding sequences (Fig. 2a) revealed that the mean (± SE) nucleotide diversity of RXLRs (1.63 × 10^−3^ (± 1.10 × 10^−4^)) was significantly higher than for the non‐RXLRs (1.14 × 10^−3^ (± 0.90 × 10^−4^)) (Mann–Whitney test, *W* = 58962.5, *P* = 0.0282 (adjusted for ties)).

An outlier analysis identified seven PITGs (PITG_06432, PITG_07947, PITG_10396, PITG_11484, PITG_13018, PITG_14986 and PITG_18325) for which the nucleotide diversity (π_i_) fell outside the 99% confidence interval (CI) of the distribution of π_i_ values, which suggests that these PITGs may be evolving non‐neutrally (Fig. 2b). The difference between the RXLR and non‐RXLR distributions of π_i_ is driven by these seven outlier loci, without which the difference in π_i_ is no longer significant (Mann–Whitney test, *W* = 55952.5, *P* = 0.0802 (adjusted for ties)). The seven outlier genes also had a significantly higher observed heterozygosity (mean(± SE) = 0.4970(± 0.1080)) compared with all targeted PITGs (mean(± SE) = 0.4287(± 0.1113), Mann–Whitney test, *W* = 32582.5, *P* = 0.0002 (adjusted for ties)). Pairwise comparisons of these seven genes in isolate 3928A with five other isolates (88069, 110059, 110153, EC1‐C7, T30‐4) revealed that one or more polymorphisms were detected in 96 of 115 pairwise comparisons (83.5%). Furthermore, in 70 of 96 comparisons (72.9%), the number of nonsynonymous substitutions exceeded the synonymous ones (Table S3). Altogether, these data suggest that the gene diversity at these seven outlier loci may be elevated relative to other PITGs by balancing selection. Intriguingly, these PITGs all encode for RXLR effector candidates annotated by Haas *et al*. (2009), and include *Avr10* (PITG_11484), a homologue of *Avr1* (PITG_06432), which is functionally distinct from *Avr1* (Du *et al*., 2015), and PexRD26 (PITG_07947), which triggers cell death in pepper (Lee *et al*., 2014).

### PenSeq reveals *P. infestans* RXLR gene presence/absence polymorphisms and allelic variation

**Figure 3:**
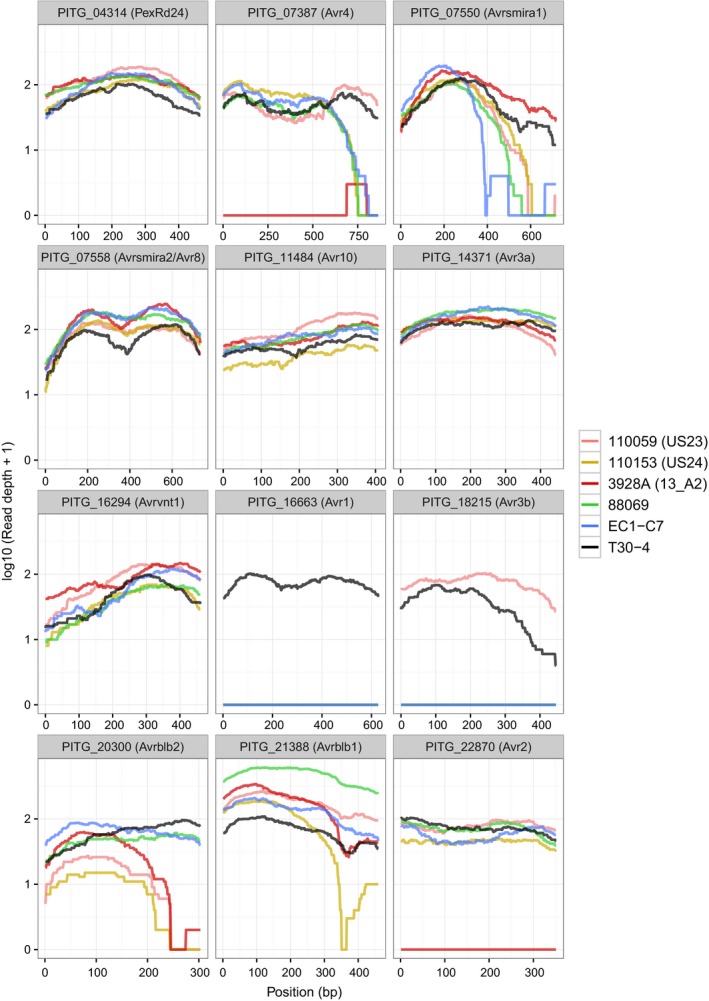
Presence/absence variations of *Phytophthora infestans Avr* genes. The *x*‐axis represents the nucleotide sequence of the full‐length reference genes *Avr1* (PITG_16663), *Avr2* (PITG_22870), *Avr3a* (PITG_14371), *Avr3b* (PITG_18215), *Avr4* (PITG_07387), *Avr8*/*AvrSmira2* (PITG_07558), *Avr10* (PITG_11484), *AvrSmira1* (PITG_07550) and *Avr_vnt1* (PITG_16294), and the *y*‐axis represents the PenSeq read coverage. PenSeq reads from the isolates T30‐4, 88069, EC1‐C7 (EC1), 3928A (13_A2), 110059 (US23) and 110153 (US24) are shown in distinct colours. Mapping of the reads is carried out at 1% mismatch rate

PenSeq reads from T30‐4, 3928A, 88069, 11059, 110153 and EC1‐C7 were used to establish simultaneously the presence/absence and allelic variation of known recognized effector (*Avr*) genes. PenSeq reads were mapped allowing a 1% mismatch rate to T30‐4 supercontigs. Genomic regions representing selected recognized effector genes were oriented 5′ to 3′ according to the gene coding sequence, and subsequently extracted (Fig. 3). The mapping data for selected genes are shown, including PITG_04314 (also known as PexRD24, a putative non‐host determinant in pepper) (Lee *et al*., 2014), PITG_07387 (*Avr4*) (van Poppel *et al*., 2008), PITG_07550 (*Avrsmira1*) (Rietman *et al*., 2012), PITG_07558 (*Avr8/Avrsmira2*) (Rietman *et al*., 2012; Vossen *et al*., 2016)*,* PITG_22870 (*Avr2*) (Gilroy *et al*., 2011), PITG_11484 (*Avr10*; K. P. Kandel, unpublished), PITG_14371 (*Avr3a*) (Armstrong *et al*., 2005), PITG_16294 (*Avrvnt1*) (Pel, 2010; Vleeshouwers *et al*., 2011), PITG_16663 (*Avr1*) (Vleeshouwers *et al*., 2011), PITG_18215 (*Avr3b*) (Rietman *et al*., 2012), PITG_20300 (*Avrblb2*) (Oh *et al*., 2009) and PITG_21388 (*Avrblb1*; IPIO1) (Vleeshouwers *et al*., 2008; Champouret *et al*., 2009). A summary of the 2345 SNPs found in all *P. infestans* PITG genes is presented in Table S4.

PenSeq reads from the six diverse isolates in this study were obtained, which correspond to the full‐length sequences of PITG_04314 (PexRD24), PITG_07558 (*Avr8*), PITG_11484 (*Avr10*), PITG_14371 (*Avr3a*) and PITG_16294 (*Avrvnt1*). This is in line with previous research which suggested that, for example, PITG_04314 and *Avr3a* are ‘core’ effectors that are likely to be present in diverse genotypes (Armstrong *et al*., 2005; Bos *et al*., 2010; Boevink *et al*., 2016). For *Avr3a*, six sequence polymorphisms were identified, four of which result in amino acid substitutions (Table 3). These include S19C in the SP, which has been described previously by Armstrong *et al*. (2005) and Cárdenas *et al*. (2011), and two amino acid changes in the C‐terminal part of *Avr3a* in positions 80 and 103 (E80K and M103I), which determine virulence/avirulence in plants containing *R3a* (Armstrong *et al*., 2005; Bos *et al*., 2006). One further amino acid substation, R124G, was found in the isolate 110059 (US23), and has been described previously in isolates derived from Mexico (Seman, 2013).

**Table 3:** Sequence diversity within selected, known *Phytophthora infestans Avr* genes

Gene name	Amino acid (s)	Cons.	Ref.	Alt.	Isolates					
					T30‐4	3928A (13_A2)	88069	110059 (US23)	110153 (US24)	EC1‐C7
*Avr3a*	S19C	NS	A	T	A	A	A	A/T	A	A
	S19S	S	C	T	C/T	C	C	C	C	C
	E80K	NS	G	A	G	G	G	G/A	G	G
	M103I	NS	G	T	G	G	G	G/T	G	G
	L121L	S	T	C	T	T	T	T/C	T	T
	R124G	NS	C	G	C	C	C	C/G	C	C
*Avr3b*	R41L	NS	C	A	C	.	.	A	.	.
	G85R	NS	C	G	C	.	.	G	.	.
	R124K	NS	C	T	C	.	.	T	.	.
*Avr4*	L139S	NS	T	C	T	.	C	T	C	C
	L221V	NS	T	G	T	.	G	T	G	G
*Avrvnt1*	P107P	S	A	G	A	A	G	A/G	G	G
*Avrblb1*	R2R	S	T	G	T/G	T/G	T/G	G	T	T/G
	L26L	S	C	A	C	C	C/A	C	C	C
	A113A	S	T	C	T	T/C	T	T	C	T
	A134G	NS	C	G	C	C/G	C	C	‐	C
	K143N	NS	A	C	A	A/C	A	A	‐	A
*Avrblb2*	R78K	NS	G	A	G	.	G/A	.	.	G
*Avr10*	D84A	NS	T	G	T/G	T/G	T	T	T/G	T
	D84N	NS	C	T	C	C/T	C	C	C/T	C/T
	D118G	NS	T	C	T	T/C	T	T	T/C	T/C
	D118N	NS	C	T	C/T	C/T	C	C	C/T	C
	K122R	NS	T	C	T	T	T	T	T	T/C
*Avrsmira1*	A44A	S	T	C	T	T	T/C	T/C	T/C	T
	P45P	S	T	A	T	T	T/A	T/A	T/A	T
	N123N	S	C	T	C	C	C	C	C	T
	K131R	NS	A	G	A	A/G	G	G	G	.
	M156L	NS	A	T	T	A/T	A	A	A	.
	L162L	S	G	A	G	G	.	A	G	G
	R170Q	NS	G	A	G	G	.	.	A	.
*PexRd24*	N18D	NS	A	G	A	A	A	A/G	G	A/G

The sequence diversity of selected known *P. infestans Avr* genes is shown in isolates T30‐4, 3928A (13_A2), 88069, 110059 (US23), 110153 (US24) and EC1‐C7 (EC1). The nucleotide sequence variations (Alt.) compared with the reference allele (Ref.), which refers to the T30‐4 genome sequence from Haas *et al*. (2009), are shown. The consequence (Cons.) of the variation is marked: S, synonymous polymorphism; NS, nonsynonymous variation. Sites that failed the single nucleotide polymorphism (SNP) filtering parameters are shown as (‐). (.), absence of sequences at the site and therefore the absence of polymorphisms.

By contrast, PITG_18215 (*Avr3b*) was only represented by PenSeq reads from the isolates T30‐4 and 110059 (US23). In these two isolates, three nonsynonymous substitutions were identified (R41L, G85R and R124; Table 3). PITG_16663 (*Avr1*) was only identifiable in the T30‐4 reference genome and is identical to the nucleotide sequence described by Haas *et al*. (2009). The absence of conserved sequences that represent *Avr3b* and *Avr1* in 3928A corroborates the whole‐genome sequence analysis described by Cooke *et al*. (2012).

PITG_20300 and PITG_21388 are representative RXLRs of the highly diverse, multigenic *Avrblb2* and *Avrblb1* families, respectively. PITG_20300 was highly conserved in the isolates T30‐4, EC1‐C7 and 88069 (Fig. 3). In addition, a heterozygous nonsynonymous substitution, R78K, was identified in the isolate 88069, which has been reported previously, but has no associated function (Oliva *et al*., 2015; Table 3). PITG_20300 is only partially represented in isolates 3928A, 11059 and 110153 (Fig. 3).

PITG_21388 (*Avrblb1*), which is also known as IPIO, is part of a highly diverse gene family, and > 16 sequence variants (in three distinct phylogenetic classes) have been described following PCR amplification in diverse isolates (Champouret *et al*., 2009). However, despite this diversity, the gene family is represented by a single gene model in the T30‐4 genome sequence (PITG_21388). IPIO class I variants are important for recognition and subsequent resistance by *Rpi‐blb1* (Champouret *et al*., 2009). All isolates, with the exception of 110153, contained full‐length PITG_21388 (Fig. 3). In total, five polymorphisms were identified at 1% mismatch in PITG_21388, two of which resulted in amino acid substitutions (A134G, K143N; Table 3), both of which have been well documented previously (Champouret *et al*., 2009).

By using the PCR amplicon sequences identified by Champouret *et al*. (2009) as references for the additional IPIO family members, and a read mapping protocol that effectively does not allow mismatches, the isolates 88069, 3928A, 110153 and EC1‐C7 were determined to have full sequence representation of the known IPIO class I variant O10, and isolates 3928A, EC1‐C7 and 110059 of the IPIO class I variant O11 (Fig. S4b). Intriguingly, although these IPIO variants had not been described in the T30‐4 reference genome, we detected almost complete sequence representation of IPIO class I members O10/O11, as well as IPIO class II members O13/O3, in T30‐4‐derived PenSeq reads (Fig. S4b).

### PenSeq enables the detection of allelic variants/paralogues of *Avrblb1* (IPIO) *and Avrblb2*

The realization that variants of *Avrblb1*, which had been identified by PCR from diverse isolates but not in the T30‐4 genome, have almost complete coverage by T30‐4‐derived PenSeq reads (Fig. S4b) prompted us to test whether PenSeq data could be used to deduce the sequences of such highly polymorphic RXLR gene variants. A key concept utilized for this *in silico* variant analysis was the identification of uni‐reads. Here, we use the strictest sense of a uni‐read to describe reads that Bowtie 2 was unable to map to any alternative map position in the reference genome at the prescribed mismatch rate.

For *Avrblb1* (PITG_21388 and associated PCR variants), the sequence identity ranges between 87% and 99%. We reduced the score‐min threshold to allow an incremental mapping of reads with up to 4% polymorphic nucleotides. Subsequently, we subtracted reads that were identical to the reference from the resulting SAM files, revealing clear evidence of allelic/paralogous variation of *Avrblb1* in T30‐4. Critically, all reads that mapped to this locus were uni‐reads. The read depth afforded by enrichment sequencing was sufficient to enable haplotypes for the variants to be determined *de novo* (Fig. S5).

Using this approach, we determined three variants of PITG_21388 in T30‐4, which we designated as A1, A2 and A3 (Table S5). Comparison of these predicted sequences with the IPIO PCR sequences obtained by Champouret *et al*. (2009) revealed that A2 was identical to IPIO class I member O10 and A3 was identical to IPIO class II member O3 over the 396 bp of comparable sequence (Fig. S6). As we mapped to the T30‐4 genome, we were able to predict additional SNPs in the remaining 5′ coding sequence not covered by the PCR products (459 bp in total) and flanking regions (Fig. S6; Table S5). We extended the analysis to the other five *P. infestans* isolates and found evidence for PITG_21388_A2 or PITG_21388_A2‐like sequences in all of these isolates (Table S5). We then generated new sequence references for the *de novo* predicted PITG_21388 variants and mapped the PenSeq reads from all isolates back to these at a 0% mismatch (Fig. S7). In line with our prediction and in agreement with Champouret *et al*. (2009), we achieved full sequence coverage of new variants across different isolates (Table S6). The data are consistent with an expansion of what had previously been thought of as a single copy gene to a small gene family with allelic variants. The *de novo* prediction of IPIO variants provides, in this case, an independent confirmation of the IPIO haplotypes defined by Champouret *et al*. (2009).

Coding sequences of the seven members of the *Avrblb2* family vary in nucleotide identity from 96% to 100%. Consequently, reducing the score‐min threshold to allow reads to map at a 4% mismatch rate inevitably runs the risk of mapping reads to a locus which, in fact, originates from family members. However, by using uni‐reads derived at different mismatch rates (Table S7), we were able to predict five allelic variants for four of the seven family members (Table S5; Fig. S8). Mapping the PenSeq reads to each member of this gene family at 0% mismatch (Fig. S4a) surprisingly suggested that none of the six *P. infestans* isolates, including T30‐4, contained full coverage of PITG_18683. However, examination of the uni‐reads that mapped to this locus at between 1% and 4% mismatch rates (Table S7) revealed evidence for the same polymorphic sequence in all reads derived from T30‐4. We subsequently refer to this variant form as PITG_18686_T30‐4 (Table S5). Additional variants were identified for PITG_04090, PITG_20300 and PITG_20303 (Table S5). As with *Avrblb1*, we generated a unique reference for these sequence variants and re‐mapped all PenSeq reads from the six isolates at a 0% mismatch rate to these bespoke references. The error‐corrected sequence, PITG_18683_T30‐4, was 100% identical and fully represented in the isolates T30‐4, 88069 and EC1‐C7, which indicates the presence of the *de novo* discovered sequence variant (Table S5) in these isolates (Fig. S9; Table S8).

### Mapping PenSeq reads to *P. infestans* genome scaffolds identifies additional effector candidates

The prediction of putative bait annealing sites that exhibit a minimum of 80% sequence identity to the *P. infestans* and *P. capsici* baits was further investigated through the mapping of T30‐4‐derived PenSeq reads. This analysis enabled the identification of 41 genomic intervals for which open reading frames (ORFs) could be established that potentially encode for previously un‐annotated RXLRs (Whisson *et al*., 2007; Haas *et al*., 2009). Amongst the 41 ORFs, 11 overlap at least partially (including on the complementary strand) with genomic regions denoted as candidate genes by PITG identifiers, but which were not annotated as RXLR effectors. The remaining 30 ORFs do not correspond to genes previously predicted (absence of PITG identifier) in the T30‐4 reference genome (Table S9). Of these 41 ORFs, 18 were specifically targeted by baits designed from *P. capsici* RXLR effectors PcRXLR038, PcRXLR078, PcRXLR195, PcRXLR271, PcRXLR272, PcRXLR294 and PcRXLR352 (Table S9). Baits from PcRXLR195 enabled the enrichment of eight distinct regions in the T30‐4 genome and yielded homologous sequences that contain signal SPs and RXLR sequences (two examples are detailed in Fig. S10). Similarly, PcRXLR271 facilitated the enrichment of four RXLR‐containing genes in the T30‐4 genome (Table S9).

Some of the newly predicted SP‐RXLR‐containing ORFs are short and thus may be pseudogenes (Table S9). For example, six of the 41 predicted ORFs were smaller than 60 amino acids and, in total, 22 were smaller than 80 amino acids. Evidence for gene expression was sought for the predicted 41 ORFs by mapping reads from 34 publicly available RNA sequencing (RNA‐Seq) datasets involving *P. infestans* studies from the Sequence Read Archive (Table S10). Of the 41 ORFs predicted following PenSeq enrichment, 17 were expressed in at least three sets and 12 in at least five experiments. The latter included nine effector candidates identified following cross‐hybridization with *P. capsici*‐derived probes (Tables S9, S10).

## Discussion

Current *Phytophthora* population studies often utilize co‐dominant, simple sequence repeat (SSR) markers, including a set of 12 SSR markers developed for *P. infestans* by Lees *et al*. (2006). These neutral markers are well established worldwide and enable the rapid genotypic classification of isolates as part of population genetic studies (Widmark *et al*., 2011; Delgado *et al*., 2013; Fry *et al*., 2013; Childers *et al*., 2015; Tian *et al*., 2016). SSR markers are useful for the study of demographic processes, and can reveal population bottlenecks and founder events, as well as patterns and rates of gene flow. However, because of their selective neutrality, SSR markers have limited utility in the analysis of adaptive evolution, and only through genetic hitchhiking can they reveal selection in neighbouring genomic regions (i.e. selective sweeps and background selection) (Casa *et al*., 2005). Hence, the correlation between genotypic variation and phenotypic variations in virulence, aggressiveness, host adaptation and race (i.e. the ability to overcome deployed resistance genes) often remains elusive (Delgado *et al*., 2013). Population genomic studies offer a way forward, using whole‐ or reduced representation genome sequencing across natural pathogen populations to identify genes that influence disease development (Grünwald *et al*., 2016). PenSeq provides a focused, reduced genome representation sequencing method which can address complex biological questions on a population scale. From a population biology and evolutionary perspective, PenSeq offers two important advantages over traditional neutral markers, such as SSRs. First, by targeting hundreds of genes and thousands of SNPs across the genome, population genetic summary statistics, such as *F*_ST_, can be estimated with little or no bias, using sample sizes as small as *N* = 4 per population (Willing *et al*., 2012). Second, by targeting effector genes, genetic variation that is under positive (or balancing) selection can be studied directly, enabling the identification of causal genetic variants, and revealing patterns of adaptive evolution with higher statistical power and lower type I error rate (false positive rate) than can whole‐genome sequencing approaches. Indeed, the analysis identified seven RXLRs amongst 433 PITG genes (259 RXLRs and 174 non‐RXLRs) that had PenSeq representation across all six *P. infestans* isolates (Fig. 2b). These seven loci displayed a significantly elevated nucleotide diversity (π_i_) and observed heterozygosity compared with all targeted PITGs, which is consistent with balancing selection. Positive selection can also increase gene diversity when a novel, favourable variant increases in frequency in the population. However, this effect is transient, because positive selection will ultimately cause the fixation of the favoured variant, thus reducing polymorphism of the gene under selection and its nearby genomic region (cf. a selective sweep; Biswas & Akey, 2006). Hence, we postulate that balancing selection, such as novel allele or rare allele advantage (Phillips *et al*., 2018), is most consistent with the pattern of polymorphism at these seven PITG genes. In a companion paper, we show the population genetic application of PenSeq employed on field samples (Jouet *et al*., 2019).

The availability of high‐throughput and accurate sequencing technologies has enabled whole‐genome sequencing of different *Phytophthora* species (reviewed in Grünwald, 2012). Moreover, several isolates of *P. infestans* have been sequenced (Haas *et al*., 2009; Cooke *et al*., 2012; Martin *et al*., 2013, 2016; Yoshida *et al*., 2013), providing an early insight into the diversity of this pathogen. However, genome assemblies are complex and the read coverage for genes of interest can be quite low. This is particularly problematic when disentangling allelic variants from CNV in multigene families, which requires (ultra)deep sequencing (Lighten *et al*., 2014), and can lead to insufficient sequence coverage to accurately determine SNPs across a population. This holds especially true for *P. infestans,* which has the largest oomycete genome to date (~240 MB) (Haas *et al*., 2009), and for which repetitive elements account for *c*. 74% of the genome (Haas *et al*., 2009). Target enrichment and sequencing provide a cost‐effective alternative to whole‐genome sequencing, and have been used previously to sequence gene families and reduce genome complexities (Mamanova *et al*., 2010). Furthermore, with regard to hypothesis testing, targeted sequencing is superior to whole‐genome sequencing because it reduces the type I error rate (false positive rate), thereby increasing the statistical power.

In this study, PenSeq was applied to pathogen genes, such as effectors, which are predicted to provide information relating to host–pathogen coevolution. PenSeq can thus be used to inform population genomic studies related to disease development and resistance gene efficacy, which could benefit and direct breeding programmes, disease control strategies and resistance deployment strategies. Critically, PenSeq reduced the genome complexity of *P. infestans* from 240 MB to < 0.5 MB (*c*. 0.2% of the genome). For *P. capsici*, the gene space of interest was 0.394 MB of a total of 64 MB for the genome, which represents *c*. 0.62% of the genome or a > 162‐fold complexity reduction. We multiplexed six *P. infestans* and six *P. capsici* strains in a single PenSeq experiment and, owing to genome reduction, achieved full representation for over 87% of all specifically re‐sequenced genes from the T30‐4 and LT1534 reference genomes at an average read depth of 70 and 85, respectively. The remaining, partially covered genes displayed > 86% sequence representation (Fig. 1). We could corroborate by PCR the absence of selected effectors in the genome of T30‐4 and other isolates, which suggests a genuine presence/absence polymorphism, rather than ambiguity in the PenSeq approach. Indeed, such deletions of genomic segments in *P. infestans* are not uncommon and have been reported previously (Van Der Lee *et al*., 2001; Jiang *et al*., 2006). It is also worthwhile noting that this specific isolate of T30‐4, studied by PenSeq, has been maintained for over 10 yr and has lost its ability to infect normally susceptible hosts, such as potato cultivar Craig Royal and the model Solanaceae *Nicotiana benthamiana*. As the enrichment on all isolates was performed simultaneously, and the effectors that were absent from T30‐4 could be identified in other isolates, we conclude that the enrichment approach is highly robust and representative.

Typically, more than one‐half of the PenSeq reads that could be mapped to the reference genomes were within the predicted bait binding sites and thus allowed a marked increase in the read depth achieved for the target genes when compared with the expected value of an untargeted whole‐genome sequencing approach. These on‐target rates are comparable with those reported in other enrichment sequencing studies, such as Van Weymers *et al*. (2016), where on‐target rates ranged from 50% to 70%. For the large genome of *P. infestans*, for example, > 300‐fold enrichment was achieved and, for the smaller *P. capsici* genome, a 100‐fold increase in read depth was observed. This makes PenSeq highly cost‐effective compared with non‐enriched whole‐genome sequencing, as consequently 300 or 100 times more raw sequencing data would be required, respectively, to achieve the same read depth for effector analysis. RNA‐Seq, which also represents a genome reduction as only expressed genes are sequenced, is less suitable for high‐throughput evolutionary genomics studies as, typically, multiple time points, pre‐ and post‐infection, are required for sequencing to ensure the representation of all known effectors (Wang *et al*., 2011). This, in turn, increases the costs significantly compared with PenSeq. Similarly, PCR‐based analysis of effector diversity is significantly more labour intense and slower as, for example, shown for *P. infestans Avr2* (Gilroy *et al*., 2011). Crucially, PenSeq evaluates all known effectors simultaneously, whereas a PCR‐based analysis is limited to single genes per reaction. PenSeq can thus be deployed to investigate specific, targeted genomic variation across a large‐scale population study at relatively low cost, and to help to identify candidate genes for functional characterization via association genetics. In addition, it could be applied to segregate populations resulting from crosses to identify loci that explain phenotypic variation, as well as for population and evolutionary genetic analyses.

PenSeq was used to perform a presence/absence avirulence (*Avr*) effector analysis (Fig. 3) and detailed corresponding SNP analysis (Tables 3, S4) for *P. infestans*. Both types of analysis corroborated previously described patterns of *Avr* diversity that were established by whole‐genome sequencing and/or PCR‐based allele mining (Vleeshouwers *et al*., 2011), but did so simultaneously for all six *P. infestans* isolates and all genes of interest. Thus, in addition to applications in population genomics to identify candidate genes that explain phenotypic variation, PenSeq can be used to predict virulence or avirulence. For example, whereas *Avr4* is present in the T30‐4 genome, indicating that the cognate resistance gene R4 would provide resistance to it, no functional *Avr4* is present in the 3928A genome, indicating that this isolate of *P. infestans* evades R4 resistance. In the future, monitoring *Avr* gene diversity across a population could inform on which *R* genes to deploy, and on whether a deployed *R* gene would be vulnerable to becoming ineffective.

The read depth achieved through PenSeq, combined with a selection of uni‐reads that were specific to a single gene and its variants, enabled us to identify novel forms for the complex *Avrblb1* and *Avrblb2* family which, on inspection, were present in multiple isolates (Figs S7, S9). For example, we predicted *de novo* variants of the *Avrblb1* effector PITG_21388, which turned out to be identical to variants previously mined by PCR (Champouret *et al*., 2009). This makes it likely that allelic/paralogous variation in RXLRs has been understudied, even in the reference strain T30‐4.

Furthermore, PenSeq data enabled us to correct a gene model for the *Avrblb2* member PITG_18683 (referred to as PITG_18683*_*T30), which we detected in the isolates T30‐4, 88069 and EC1‐C7. The original gene model for PITG_18683 was derived from the T30‐4 genome, but was absent in all isolates at a 0% mismatch rate. The plasticity of the *P. infestans* genome, in terms of the tendency to delete effectors, is apparent, with isolates lacking subsets of the *Avrblb2* family, a phenomenon which may suggest functional redundancy within the group, given that all *P. infestans* isolates tested are avirulent on plants expressing *Rpi‐blb2*.

Although not the focus of this PenSeq study, other research has demonstrated that the gene coverage depth from whole‐genome sequencing can be used to infer CNVs in genomes, including genomes from plant pathogens, beyond presence/absence CNV (Brynildsrud *et al*., 2016; Arsenault‐Labrecque *et al*., 2018). Additional, recent studies have further revealed that targeted enrichment sequencing reads are also suitable for CNV analysis (Ellingford *et al*., 2017), which provides additional scope for future applications of PenSeq technology beyond the presence/absence CNV analysis described here.

The examination of regions of the *P. infestans* T30‐4 genome that yielded a large number of PenSeq reads, but for which no PITG gene models were described, revealed a correlation in sequence homology to other *P. infestans*‐ or *P. capsici*‐specific RXLR‐derived probes. A search for ORFs and canonical SP‐RXLR domains corroborated that the gene models that were consequently established displayed high homology to characterized RXLR‐type effectors (Table S9; Fig. S10), several of which had only previously been annotated in the *P. capsici* genome. Thus, it was hypothesized that some putative RXLR‐encoding genomic regions had been omitted in the original *P. infestans* genome annotation.

It is likely that the complement of RXLR effectors has been underestimated in *P. infestans*. This is important for studies aiming to identify *Avr* candidates recognized by new R proteins using effectoromic studies (Vleeshouwers *et al*., 2011). Importantly, some of the newly identified *P. infestans* RXLR candidates have been identified as a consequence of the achieved enrichment with probes thought to be specific for *P. capsici*. Given that these pathogens share some common hosts (e.g. tomato), as well as infecting distinct hosts (e.g. potato and pepper), the analysis of RXLR effectors that are shared or distinct between *P. infestans* and *P. capsici* will be important in understanding the host range and non‐host resistance. This also shows that the use of bait targeting designs in related species, rather than a single species, helps to identify previously unannotated genes.

In conclusion, PenSeq provides a cost‐effective approach to enrich specific portions of a pathogen or microbe genome to rapidly and accurately assess presence/absence and sequence polymorphisms across multiple individuals. The method has broad applications and can be adapted to diverse microbes and pathogens across a wide host spectrum. Used on a population scale, PenSeq reveals recognized effector genes that can determine the potential durability of a deployed crop resistance gene. Moreover, PenSeq facilitates re‐annotation of effector candidates across the *P. infestans* genome. Critically, the approach will facilitate cost‐effective, statistically powerful population genomic studies, opening the door to association studies that will help to determine the genes underlying pathogenicity, race, aggressiveness and host adaptation.

## Author contributions

GJAT and MRA performed target enrichment sequencing. GJAT, MRA, T‐YL and KB conducted computational analysis. AJ, JDGJ, EH, PRJB and IH designed the bait library. BW, CvO and GJAT conducted population genomics studies. GJAT, T‐YL, PRJB, JDGJ, MRA and IH wrote the manuscript. IH planned and designed the research.

## Supporting information

Please note: Wiley Blackwell are not responsible for the content or functionality of any Supporting Information supplied by the authors. Any queries (other than missing material) should be directed to the *New Phytologist* Central Office.

**Fig. S1** Representation of target gene coverage in *Phytophthora infestans* reference strain T30‐4 and *P. capsici* reference strain LT1534 at 2% and 5% mismatch mapping rates.
**Fig. S2** PCR amplifications of effectors and no‐template control (control) across six isolates of *Phytophthora infestans*.
**Fig. S3** Representation of target gene coverage in *Phytophthora infestans* isolates 88069, EC1‐C7, 3928A, 110059 and 110153, as well as *P. capsici* isolates LT123, LT6536, Pc204, Y006 and Q108, at a 1% mismatch mapping rate.
**Fig. S4** Presence/absence variations of complex *Phytophthora infestans Avrblb2* and *Avrblb1* family members.
**Fig. S5** Graphical representation of a sequence alignment map of T30‐4‐derived PenSeq uni‐reads mapped to PITG_21388.
**Fig. S6** Graphical sequence comparison between the reference *Avrblb1* (PITG_21388), *de novo* predicted variants A1, A2, 88069_A2 and A3, and IPIO haplotypes amplified by Champouret *et al*. (2009).
**Fig. S7** Presence/absence variations of PITG_21388 and *de novo* predicted *Phytophthora infestans* family members.
**Fig. S8** Graphical sequence comparison between reference sequence of *Avrblb2* members alongside *de novo* predicted variants.
**Fig. S9** Presence/absence variations of *de novo* predicted *Avrblb2* members.
**Fig. S10** Graphical representation of novel RXLRs identified in the T30‐4 *Phytophthora infestan*s reference genome via *P. capsici*‐derived bait hybridization.
**Methods S1** Supporting materials and methods.

**Table S1** *Phytophthora infestans* and *P. capsici* genes selected for target enrichment sequencing.
**Table S2** PenSeq read data analyses for *Phytophthora capsici* and *P. infestans* isolates.
**Table S3** The numbers and rates of synonymous and non‐synonymous substitutions based on a set of codon‐aligned nucleotide sequences of seven RXLR effectors that showed a significantly elevated level of nucleotide diversity and heterozygosity.
**Table S4** Sequence diversity within known *Phytophthora infestans* genes (PITGs) for all six *P. infestans* isolates.
**Table S5** Sequence diversity within *de novo* predicted *Avrblb2* and *Avrblb1* variants for all six *Phytophthora infestans* isolates.
**Table S6** The percentage coverage of full‐length coding sequence for PITG_21388, *de novo* predicted *Phytophthora infestans* family members A1, A2 and A3 in T30‐4, and an 88069 specific form of A2.
**Table S7** Number of uni‐reads identified for *Avrblb2* members in the isolates T30‐4, 88069, EC1‐C7 (EC1), 3928A (13_A2), 110059 (US23) and 110153 (US24) as a function of relaxing the mismatch rate for the PenSeq read mapping from 0%, 1%, 2%, 3% and 4%.
**Table S8** The percentage coverage of full‐length coding sequence for *Avrblb2* family members and *de novo* predicted variants.
**Table S9** *Phytophthora infestans*‐ and *P. capsici*‐derived bait hybridization identifies novel RXLRs in the T30‐4 *P. infestans* reference genome.
**Table S10** Expression analysis of bait hybridization‐based predicted RXLRs.
