# Supplementary material for: Pathogen enrichment sequencing (PenSeq) enables population genomic studies in oomycetes
Source: New Phytol. 2018 Oct 5;221(3):1634–48. doi: 10.1111/nph.15441 (PMC6492278; doi:10.1111/nph.15441)
Supplement: Supplementary file 1 — Fig. S1 Representation of target gene coverage in Phytophthora infestans reference strain T30‐4 and P. capsici reference strain LT1534 at 2% and 5% mismatch mapping rates. Fig. S2 PCR amplifications of effectors and no‐template control (control) across six isolates of Phytophthora infestans. Fig. S3 Representation of target gene coverage in Phytophthora infestans isolates 88069, EC1‐C7, 3928A, 110059 and 110153, as well as P. capsici isolates LT123, LT6536, Pc204, Y006 and Q108, at a 1% mismatch mapping rate. Fig. S4 Presence/absence variations of complex Phytophthora infestans Avrblb2 and Avrblb1 family members. Fig. S5 Graphical representation of a sequence alignment map of T30‐4‐derived PenSeq uni‐reads mapped to PITG_21388. Fig. S6 Graphical sequence comparison between the reference Avrblb1 (PITG_21388), de novo predicted variants A1, A2, 88069_A2 and A3, and IPIO haplotypes amplified by Champouret et al. (2009). Fig. S7 Presence/absence variations of PITG_21388 and de novo predicted Phytophthora infestans family members. Fig. S8 Graphical sequence comparison between reference sequence of Avrblb2 members alongside de novo predicted variants. Fig. S9 Presence/absence variations of de novo predicted Avrblb2 members. Fig. S10 Graphical representation of novel RXLRs identified in the T30‐4 Phytophthora infestans reference genome via P. capsici‐derived bait hybridization. Methods S1 Supporting materials and methods. [file NPH-221-1634-s001.doc]

*New Phytologist* Supporting Information

# Article title: **Pathogen enrichment sequencing (PenSeq) enables** population genomic studies in oomycetes

Authors: Gaetan JA Thilliez1^,2^, Miles Armstrong^1^, Tze Yin Lim^3^, Katie Baker^3^, Agathe Jouet^4^, Ben Ward^5^, Cock van Oosterhout^6^, Jonathan DG Jones^4^, Edgar Huitema^2^, Paul RJ Birch^1,2^ and Ingo Hein^1,2*^

Article acceptance date: 13 August 2018

# Method S1

## SNP calling

The paired-end reads were mapped against the *Phytophthora infestans* T30-4 genome (Haas *et al.,* 2009) with Bowtie 2 version 2.2.9 (Langmead and Salzberg 2012) at a 1% mismatch rate (score-min “L,0,-0.06”) and the primary alignments were retained (-F 256). Duplicated reads were removed with rmdup in samtools version 1.3.1 (Li *et al.,* 2009). Variants were called with Freebayes v0.9.20 (Garrison and Marth 2012) from the bam file of each isolate with options --ploidy 2, --no-populations-priors, --report-monomorphic and --min-mapping-quality 2. The resulting VCF files were subjected to filtering with vcffilter (https://github.com/vcflib/vcflib#vcffilter) using the following settings: DP > 10 & MQM > 20 & SAF > 1 & SAR > 1 & QUAL / AO > 20 or NUMALT = 0. Variants that passed the filtering were merged into the final VCF file with vcf-merge and annotated with SnpEff V4.2 (Cingolani *et al.,* 2012) using a pre-built *Phytophthora infestans* annotation database, ASM14294v1.29. The variants that overlapped the positions of 12 known avirulence genes were obtained with BEDTools v2.25.0 (Quinlan and Hall 2010).

**Nucleotide diversity and heterozygosity study**

Corresponding PITG sequences for the 6 *P. infestans* isolates were generated using the GATK FastaAlternateReferenceMaker utility (Broad Institute 2016). Single nucleotide polymorphisms (SNPs) were detected in comparison to the T30-4 reference genome and used to replace the reference sequences in each isolate. These alternate sequences were then used to calculate nucleotide diversity (*π*_i_), as well as observed heterozygosity (*Ho*). Only genes that were present in all 6 *P. infestans* isolates were used, reducing the sample size to 433 genes (259 RXLRs and 174 non-RXLRs).

Nucleotide diversity and heterozygosity were calculated by first loading the sequences of a given gene from each isolate into *R* using the Analysis of Phylogenetics and Evolution (APE) package (Paradis *et al.,* 2004). Nucleotide diversity was calculated using the nuc.div function of the Population and Evolutionary Genetics Analysis System (PEGAS) package (Paradis 2010). The sequence data was then converted to a ‘genind’ and a ‘loci’ data-class to calculate heterozygosity in PEGAS. Heterozygosity values were computed per variable locus and grouped on a per transcript basis before being written to tables for plotting in SigmaPlot version 10. Differences in nucleotide diversity and heterozygosity between the RXLRs and non-RXLRs were tested using a Mann-Whitney Tests. Nucleotide diversity (*π*_i_) values that fell outside the distribution of predicted values (5 – 95% CI) were identified using an outlier analysis that simulated the null distribution of π_i_ based on a mutation-drift equilibrium using the software pgcalc (https://github.com/BenJWard/pgcalc). The numbers and rates of synonymous and non-synonymous substitution based on a set of codon-aligned nucleotide sequences of 7 RXLR effectors with significantly elevated levels of nucleotide diversity and heterozygosity were calculated using the software SNAP 2.1.1 (Korber 2001). Statistical analyses were performed in Minitab 17.

***De novo* RXLR prediction**

For the prediction of additional RXLR around bait binding sites, Bedtools (Quinlan and Hall 2010) was used to identify ORF with a direct overlap with predicted bait binding site. The ORFs were queried for the presence of a signal peptide using SignalP versions 3 (Bendtsen *et al.,* 2004) and 4 (Petersen *et al.,* 2011) and then further evaluated for the occurrence of RXLR motifs with a Galaxy pipeline (Cock *et al.,* 2013), which utilises complementary algorithms developed by Bhattacharjee *et al.,* (2006), Win *et al.,* (2007) and Whisson *et al.,* (2007).

**Relative gene expression quantification of additional candidate RXLRs**

Three independent RNA-seq dataset with 34 individual samples were downloaded from the Sequence Read Archive (accessions: PRJEB12248, PRJNA266335 and PRJNA303084). The raw reads were first assessed with FastQC v0.11.5 (Andrews 2010) and trimmed with fastq-mcf 1.04.676 (Aronesty 2013) to retain reads with no adapters, a minimum base quality of 28 and a minimum length of 76 bp. The resulting trimmed reads were mapped to the reference genome of *P. infestans* T30-4 (Haas *et al.,* 2009) with hisat2-2.0.4 (Kim *et al.,* 2015) using the appropriate strand specificity options (default for reads in PRJEB12248, --rna-strandness RF for the paired-end reads in PRJNA303084 and --rna-strandness R for the single-end reads in PRJNA266335). A 1% mismatch rate was set (score-min “L,0,-0.06”) and reads with mixed or discordant mapping were supressed from the output (--no-mixed, --no-discordant).

The main aim of this analysis was to quantify the relative gene expression of the candidate RXLRs within a sample, rather than to quantify differential gene expression between samples. HTSeq-0.6.1p1 (Anders *et al.,* 2015) was used to count the number of reads that mapped to the regions that were associated with the 41 candidate RXLRs in intersection-nonempty mode. All candidate RXLRs contained a single exon only. The raw counts from the mapped read were converted to Transcripts per Million (TPM, (Wagner *et al.,* 2012)) with count_to_TPM.R and log2 transformed in RStudio version 0.99.903 (RStudio Team, 2015). A candidate RXLR was considered to be putatively expressed within a sample if it has a log2 (TPM) > 2.

$$TPM=\frac{N_{i}}{L_{i}} . \left( \frac{1}{\sum_{j} \frac{N_{j}}{L_{j}}} \right) . {10}^{6}$$

Briefly, $N_{i}$ is the raw count of the reads that mapped to a gene *i* and $L_{i}$ is the length of the gene *i*. The gene fraction, $\frac{N_{i}}{L_{i}}$was computed by dividing the count of the mapped reads by the length of each gene. The sum of all gene fractions was denoted with $\sum_{j} \frac{N_{j}}{L_{j}}$.

**PCR-based validation of PenSeq predicted presence/absence variations**

We designed primers to confirm the presence/absence of 10 genes in T30-4 and other isolates.

| PITG | Forward Primer (5’-3’) | Reverse Primer (5’-3’) |
| --- | --- | --- |
| PITG_04097 | CTTCTTTCCAGTGAAATCCGTGCCTACAAG | TGTAAATTTTCTCCGCATCTGAAGCTTTATCG |
| PITG_04099 | CTCAAGTGTCAAGACCAAAAGAAACAATCCG | CCAGCGCTGAATCAAGTGTGTCGAA |
| PITG_04182 | CACGCTTGTTAGATAGCGCTCCATAGC | CCACTCTTCAATTTACTGACAAACCAAGTATCC |
| PITG_12010 | CTAAACCTCCCACCCCAGTCAGG | TTTGTACATCCACTTGAACGAGAAAGTGATG |
| PITG_16282 | CTTTCAACTCTTCAAGCGCTGCAAGC | GTTATTGATGTTGTTCAAGGTATAGCCCTTCG |
| PITG_16283 | TCGTTCAAATCATCGACGTACTTGAC | AAAAATAAAAGGTCATTTTTGATTTTCTTTCGATG |
| PITG_16285 | AGCTTAACCCTTCAAGTTAAAGAATACATCCG | AGCCTCCCACCACCATTCAGG |
| PITG_19800 | TCCCAATTTCCCCGCATCCTCAC | GGGGACATCCTTCGACGGGAC |
| PITG_22727 | GAATTTCGATTTCCTGATTCTTGATGATCTTGTTC | CCTTCTTTTAAGCGTAATCCCCCTTTTACAG |
| PITG_14371 | TAATTTCGCTCTTCACACGATAAGCTCTC | GCGTTTCAGCAGTTAGAATCGGATTTTCTG |

## Prediction of alleles/paralogues of *Avrblb1* and *Avrblb2*

PenSeq reads from the six *P. infestans* isolates were mapped to the T30-4 reference genome using bowtie2 v2.2.1 in end to end mode using four score-min thresholds per read pair; L,-0.01,-0.01 (0%), L,-0.06,-0.06 (1%), L,-0.12,-0.12 (2%), L,-0.18,-0.18 (3%) and L,-0.24,-0.24 (4% mismatch). Multimapping mode (-a) was used so all potential mapping positions for each read were reported except discordant mappings. The resulting bam files were subsampled for the CDS of the RXLRs of interest using samtools view. This resulted in bam alignments of all reads that overlaped with the coordinates of each CDS at each mismatch rate. These bam files were converted to .bed format using bedtools bamtobed v2.24.0. In the case of Avrblb1 (PITG_21388/IPIO) all mapped reads had been assigned a MAPQ score of 255 by bowtie2 reflecting the fact that no alternative mapping position had been found (all reads were uni-reads). In the case of Avrblb2, the .bed files were filtered for uni-reads using grep (grep –w “255” in.bed > out.bed). The uni-reads identified at each mismatch rate were concatenated and the resulting .bed files were converted to lists of uni-read IDs. These were used to filter the original bam files generated at the 4% mismatch rate for uni-reads using picard tools FilterSamReads (<http://broadinstitute.github.io/picard>). The resulting filtered bam alignments were visualised using Geneious R9 (https://www.geneious.com, Kearse *et al.,* 2012) and variants called manually in those instances where a variable SNP was supported by multiple uni-reads.

# Supplementary Figures

S1 Fig.


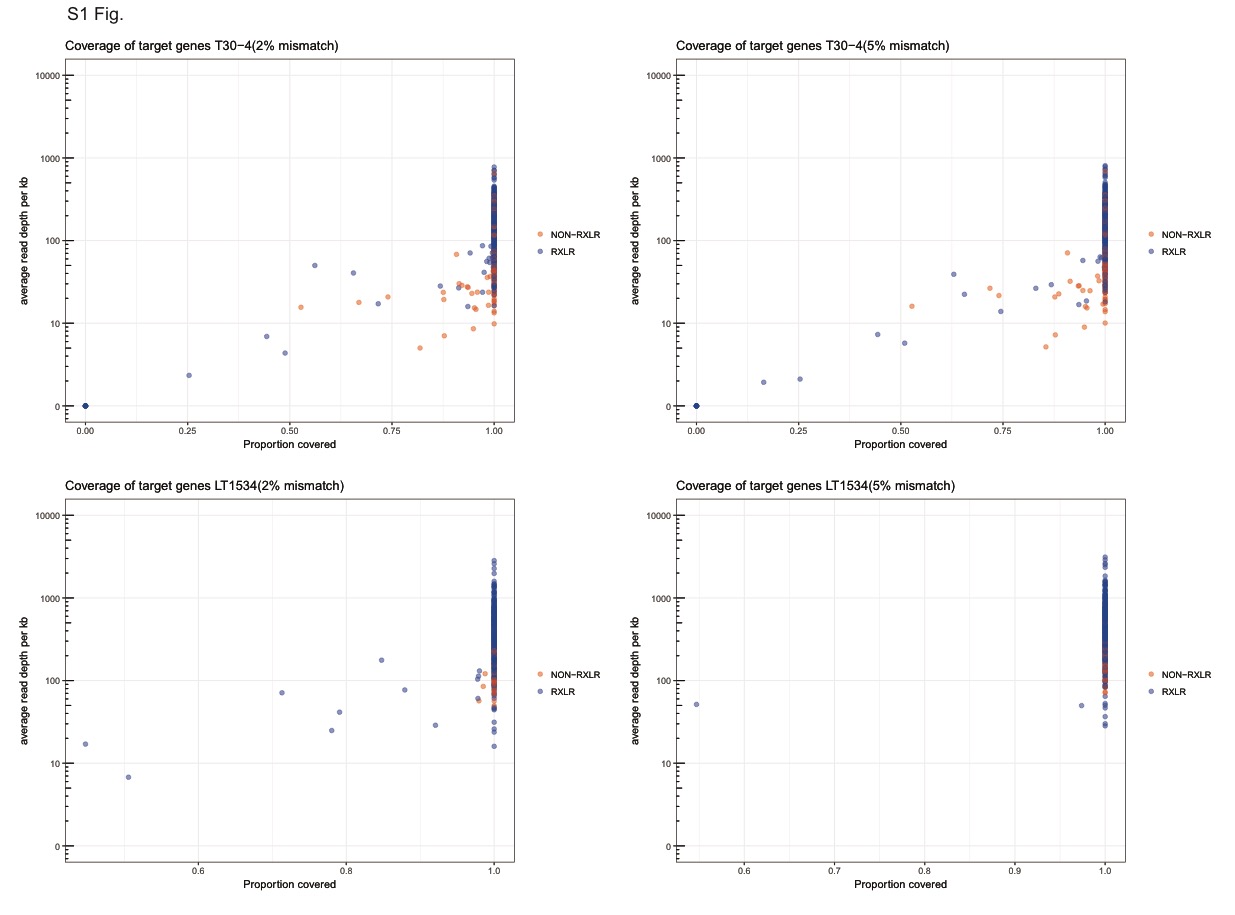


**Fig. S1:** Representation of target gene coverage in *P. infestans* reference strain T30-4 and *P. capsici* reference LT1534 at 2 % and 5% mismatch mapping rates.

The x-axis represents the percentage gene coverage of RXLRs (blue) and non-RXLR target genes (red), which ranges from 0 (not covered) to 1 (100 % sequence representation with PenSeq reads). The y-axis reveals the average read depth per kb of the target genes.

S2 Fig.

**Fig. S2:** PCR amplifications of effectors PITG_04097, PITG_04099, PITG_04182, PITG_12010, PITG_16282, PITG_16283, PITG_16285, PITG_19800, PITG_22727, positive control PITG_14371 (*Avr3a*) and no-template control (control) across six isolates (T30-4, 88069, EC1-C7 (EC1), 3928A (13_A2), 110059 (US23) and 110153 (US24)) of *P. infestans*.

S3 Fig.


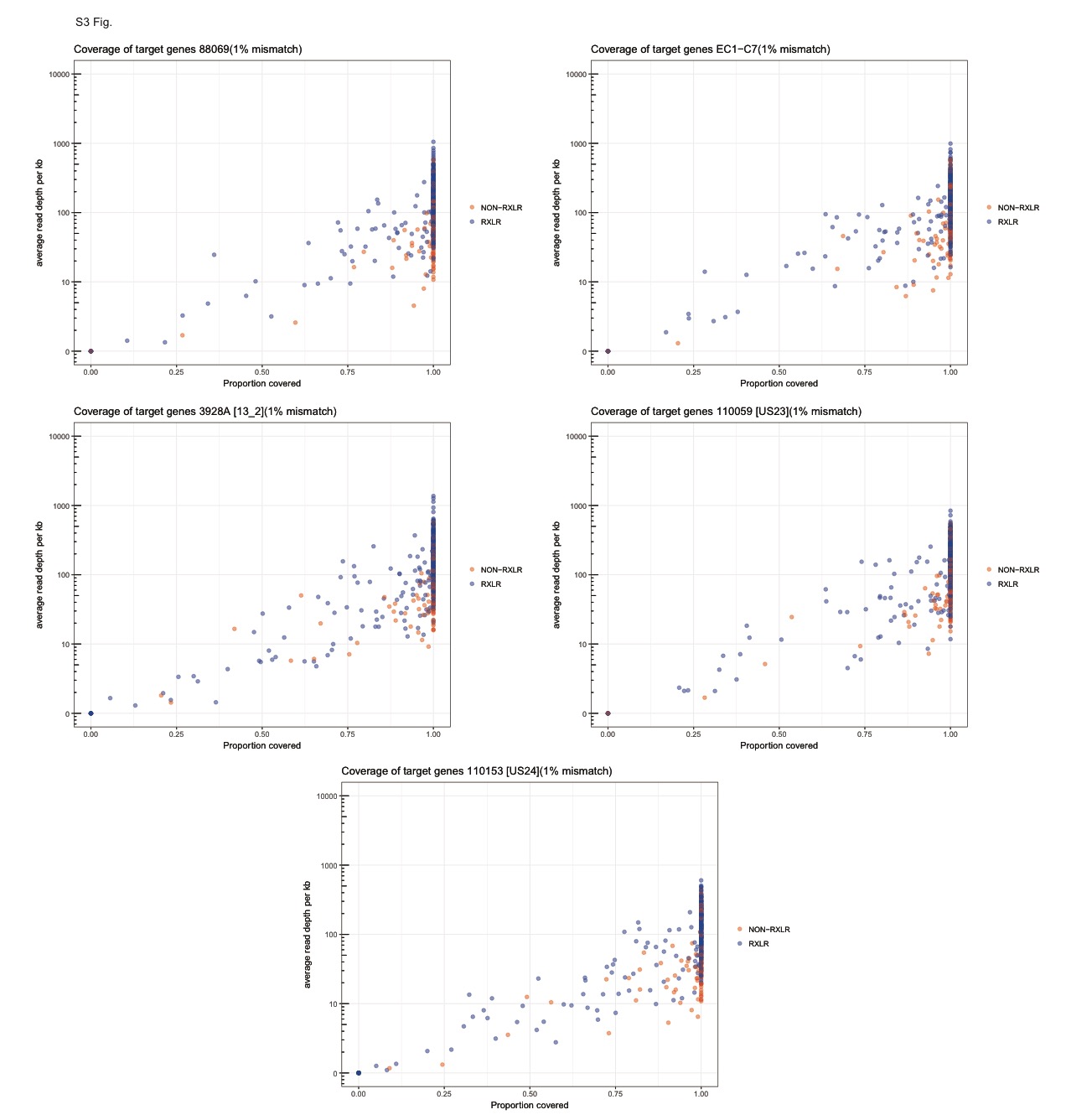


**Fig. S3:** Representation of target gene coverage in *P. infestans* isolates 88069, EC1-C7, 3928A, 110059 and 110153 as well as *P. capsici* isolates LT123, LT6536, Pc204, Y006 andQ108 at 1 % mismatch mapping rate.

The x-axis represents the percentage gene coverage of RXLRs (blue) and non-RXLR target genes (red), which ranges from 0 (not covered) to 1 (100 % sequence representation with PenSeq reads). The y-axis reveals the average read depth per kb of the target genes.

**Fig. S4:** Presence/absence variations of complex *P. infestans Avrblb2* (A) and *Avrblb1* (B) family members.

A) The x-axis represents the nucleotide sequence of the full-length *Avrblb2* members (PITG04085 (100% identical to PITG04086), PITG_04090, PITG_18683, PITG_20300, PITG_20301 and PITG_20303) obtained from the T30-4 genome and the y-axis the PenSeq read coverage. PenSeq reads from the isolates T30-4, 88069, EC1-C7 (EC1), 3928A (13_A2), 110059 (US23) and 110153 (US24) are shown in distinct colours. Mapping of the reads is carried out at a 0 % mismatch rate.

B) The x-axis represents the nucleotide sequence (based on PCR products) of the different IPIO class members as reported by Champouret *et al.,* (2009) and the y-axis the PenSeq read coverage. PenSeq reads from the isolates T30-4, 88069, EC1-C7 (EC1), 3928A (13_A2), 110059 (US23) and 110153 (US24) are shown in distinct colours. Mapping of the reads is carried out at a 0 % mismatch rate.

**Fig. S5:** Graphical representation of a sequence alignment map of T30-4 derived PenSeq uni-reads mapped to PITG_21388. Here reads that mapped at 3 % are shown after subtraction of reads that mapped at 0 % and 1 %. Sequence polymorphisms in PenSeq uni-reads compared to the reference are highlighted in colour. The reference is based on the T30-4 genome and includes flanking region within the supercontig.

**Fig. S6:** Graphical sequence comparison between the reference *Avrblb1* (PITG_21388), *de novo* predicted variants A1, A2, 88069_A2, A3 and IPIO haplotypes amplified by Champouret *et al*., (2009). Sequence polymorphisms are highlighted in colour.

**Fig. S7:** Presence/absence variations of PITG_21388 and *de novo* predicted *P. infestans* family members. The x-axis represents the nucleotide sequence of the full-length *Avrblb1* reference PITG_21388, alongside variants A1, A2 and A3 as predicted in T30-4 and a 88069 specific form of A2. The y-axis indicates the PenSeq read coverage. PenSeq reads from the isolates T30-4, 88069, EC1-C7 (EC1), 3928A (13_A2), 110059 (US23) and 110153 (US24) are shown in distinct colours. Mapping of the reads is carried out at a 0 % mismatch rate.

**Fig. S8:** Graphical sequence comparison between reference sequence of *Avrblb2* members PITG_04085, PITG_04086, PITG_04090, PITG_18683, PITG_20300, PITG_20301 and PITG_20303 alongside *de novo* predicted variants PITG_04090_3928A, PITG_04090_US24, PITG_18683_T30-4, PITG_20300_T30-4 and PITG_20303_EC1. Sequence polymorphisms are highlighted in colour.

**Fig. S9**: Presence/absence variations of *de novo* predicted *Avrblb2* members PITG_04090_110153(US24), PITG_04090_3928A(13A2), PITG_18683_T30-4, PITG_20300_T30-4 and PITG_20303_T30-4. The x-axis represents the nucleotide sequence of the full-length variants and the y-axis indicates the PenSeq read coverage. PenSeq reads from the isolates T30-4, 88069, EC1-C7 (EC1), 3928A (13_A2), 110059 (US23) and 110153 (US24) are shown in distinct colours. Mapping of the reads is carried out at a 0 % mismatch rate.

**Fig. S10:** Graphical representation of novel RXLRs identified in the T30-4 *P. infestan*s reference genome via *P. capsici* derived bait hybridisation. Shown are the regions from base pairs 1,223,590 to 1,224,268 in Supercontig 1.25 and 1,071,187 to 1,071,865 in T30-4 Supercontig 1.38. The baits, highlighted in green, are all derived for *P. capsici* RXLR effector PcRXLR195. PenSeq reads from T30-4 enrichment sequencing are mapped to the Supercontigs at a 1% mismatch rate and visualised using Tablet (https://ics.hutton.ac.uk/tablet/). The resulting T30-4 predicted Open Reading Frames (ORFs) were translated and aligned to *P. capsici* RXLR effector PcRXLR195. Highlighted are the predicted Signal Peptide and RXLR domains.

**Bibliography**

Anders S, Pyl PT, Huber W. 2015. HTSeq-A Python framework to work with high-throughput sequencing data. Bioinformatics 31:166–169.

Andrews S. 2010. FastQC: A quality control tool for high throughput sequence data. Available online at: <http://www.bioinformatics.babraham.ac.uk/projects/fastqc>

Aronesty E. 2013. Comparison of Sequencing Utility Programs. Open Bioinforma. J. 7:1–8.

Baxter L, Tripathy S, Ishaque N, Boot N, Cabral A, Kemen E, Thines M, Ah-Fong A, Anderson R, Badejoko W, *et al.,* 2010. Signatures of adaptation to obligate biotrophy in the *Hyaloperonospora arabidopsidis* genome. Science 330:1549–1551.

Bendtsen JD, Nielsen H, Heijne G Von, Brunak S. 2004. Improved Prediction of Signal Peptides : SignalP 3 . J Mol Biol. 340:783-95.

Bhattacharjee S, Hiller NL, Liolios K, Win J, Kanneganti TD, Young C, Kamoun S, Haldar K. 2006. The malarial host-targeting signal is conserved in the irish potato famine pathogen. PLoS Pathog. 2:453–465.

Champouret N, Bouwmeester K, Rietman H, van der Lee T, Maliepaard C, Heupink A, van de Vondervoort PJI, Jacobsen E, Visser RGF, van der Vossen E a G, *et al.,* 2009. *Phytophthora infestans* isolates lacking class I IPIO variants are virulent on *Rpi-blb1* potato. Mol. Plant. Microbe. Interact. 22:1535–1545.

Cingolani P, Platts A, Wang LL, Coon M, Nguyen T, Wang L, Land SJ, Lu X, Ruden DM. 2012. A program for annotating and predicting the effects of single nucleotide polymorphisms, SnpEff: SNPs in the genome of *Drosophila melanogaster* strain w 1118; iso-2; iso-3. Fly (Austin). 6:80–92.

Cock, PJ., Grüning, BA, Paszkiewicz, K, & Pritchard, L. 2013. Galaxy tools and workflows for sequence analysis with applications in molecular plant pathology. *PeerJ*, *1*, e167.

Garrison E, Marth G. 2012. Haplotype-based variant detection from short-read sequencing. arXiv Prepr. arXiv1207.3907:9.

Haas BJ, Kamoun S, Zody MC, Jiang RHY, Handsaker RE, Cano LM, Grabherr M, Kodira CD, Raffaele S, Torto-Alalibo T, *et al.,* 2009. Genome sequence and analysis of the Irish potato famine pathogen *Phytophthora infestans*. Nature 461:393–398.

Kearse, M., Moir, R., Wilson, A., Stones-Havas, S., Cheung, M., Sturrock, S., Buxton, S., Cooper, A., Markowitz, S., Duran, C., Thierer, T., Ashton, B., Mentjies, P., & Drummond, A. (2012). Geneious Basic: an integrated and extendable desktop software platform for the organization and analysis of sequence data. Bioinformatics, 28:1647-1649.

Kim D, Langmead B, Salzberg SL. 2015. HISAT: a fast spliced aligner with low memory requirements. Nat. Methods 12:357–360.

Korber B. 2001. HIV Sequence Sigmatires and Similarities. In: Rodrigo AG, Learn GH, editors. Computational and Evolutionary Analysis of HIV Molecular Sequences. Boston, MA: Springer US. p. 55–72.

Langmead B, Salzberg SL. 2012. Fast gapped-read alignment with Bowtie 2. Nat Methods 9:357–359.

Li H, Handsaker B, Wysoker A, Fennell T, Ruan J, Homer N, Marth G, Abecasis G, Durbin R. 2009. The sequence alignment/map format and SAMtools. Bioinformatics 25:2078–2079.

Paradis E. 2010. Pegas: An R package for population genetics with an integrated-modular approach. Bioinformatics 26:419–420.

Paradis E, Claude J, Strimmer K. 2004. APE: Analyses of phylogenetics and evolution in R language. Bioinformatics 20:289–290.

Petersen TN, Brunak S, von Heijne G, Nielsen H. 2011. SignalP 4.0: discriminating signal peptides from transmembrane regions. Nat. Methods 8:785–786.

Quinlan AR, Hall IM. 2010. The BEDTools manual. Genome 16:1–77.

RStudio Team (2015). RStudio: Integrated Development for R. RStudio, Inc., Boston, MA URL http://www.rstudio.com/.

Wagner GP, Kin K, Lynch VJ. 2012. Measurement of mRNA abundance using RNA-seq data: RPKM measure is inconsistent among samples. Theory Biosci. 131:281–285.

Whisson SC, Boevink PC, Moleleki L, Avrova AO, Morales JG, Gilroy EM, Armstrong MR, Grouffaud S, van West P, Chapman S. 2007. A translocation signal for delivery of oomycete effector proteins into host plant cells. Nature 450:115–118.

Win J, Morgan W, Bos J, Krasileva K V, Cano LM, Chaparro-garcia A, Ammar R, Staskawicz BJ, Kamoun S. 2007. Adaptive evolution has yargeted the C-terminal domain of the RXLR effectors of plant pathogenic oomycetes. Plant Cell. 19:2349-69
